# Etiologies of Melanoma Development and Prevention Measures: A Review of the Current Evidence

**DOI:** 10.3390/cancers13194914

**Published:** 2021-09-30

**Authors:** Amir Reza Djavid, Connor Stonesifer, Benjamin T. Fullerton, Samuel W. Wang, Marlene A. Tartaro, Bradley D. Kwinta, Joseph M. Grimes, Larisa J. Geskin, Yvonne M. Saenger

**Affiliations:** 1 Vagelos College of Physicians & Surgeons, Columbia University, New York, NY 10032, USA; ard2188@cumc.columbia.edu (A.R.D.); cjs2244@cumc.columbia.edu (C.S.); bdk2123@cumc.columbia.edu (B.D.K.); jmg2330@cumc.columbia.edu (J.M.G.); 2 Department of Medicine, Division of Hematology/Oncology, Columbia University Irving Medical Center, New York, NY 10032, USA; btf2111@cumc.columbia.edu (B.T.F.); sww2123@cumc.columbia.edu (S.W.W.); 3 Columbia College, New York, NY 10027, USA; mat2232@columbia.edu; 4 Department of Dermatology, Columbia University Irving Medical Center, New York, NY 10032, USA; ljg2145@cumc.columbia.edu

**Keywords:** melanoma, tanning, prevention, sunscreen, sun safety, screening, UV, artificial intelligence

## Abstract

**Simple Summary:**

Melanoma constitutes a major public health risk, with the rates of diagnosis increasing on a yearly basis. Monitoring for risk factors and preventing dangerous behaviors that increase melanoma risk, such as tanning, are important measures for melanoma prevention. Additionally, assessing the effectiveness of various methods to prevent sun exposure and sunburns—which can lead to melanoma—is important to help identify ways to reduce the development of melanoma. We summarize the recent evidence regarding the heritable and behavioral risks underlying melanoma, as well as the current methods used to reduce the risk of developing melanoma and to improve the diagnosis of this disease.

**Abstract:**

(1) Melanoma is the most aggressive dermatologic malignancy, with an estimated 106,110 new cases to be diagnosed in 2021. The annual incidence rates continue to climb, which underscores the critical importance of improving the methods to prevent this disease. The interventions to assist with melanoma prevention vary and typically include measures such as UV avoidance and the use of protective clothing, sunscreen, and other chemopreventive agents. However, the evidence is mixed surrounding the use of these and other interventions. This review discusses the heritable etiologies underlying melanoma development before delving into the data surrounding the preventive methods highlighted above. (2) A comprehensive literature review was performed to identify the clinical trials, observational studies, and meta-analyses pertinent to melanoma prevention and incidence. Online resources were queried to identify epidemiologic and clinical trial information. (3) Evidence exists to support population-wide screening programs, the proper use of sunscreen, and community-targeted measures in the prevention of melanoma. Clinical evidence for the majority of the proposed preventive chemotherapeutics is presently minimal but continues to evolve. (4) Further study of these chemotherapeutics, as well as improvement of techniques in artificial intelligence and imaging techniques for melanoma screening, is warranted for continued improvement of melanoma prevention.

## 1. Introduction

Skin cancer is the most common cancer type in the United States (U.S.), estimated to affect nearly one in five individuals over their lifetime [1]. Cutaneous melanoma is the most aggressive dermatologic malignancy, responsible for the vast majority of morbidity and mortality in skin cancer patients. In the United States, the yearly incidence of cutaneous melanoma is 22.8 cases per 100,000 individuals, and the disease claims the lives of 2.3 out of 100,000 individuals each year [2]. An estimated 7,180 deaths will be attributed to melanoma in 2021 [3], downtrending from the 9,128 deaths reported in 2011 [4]. However, the incidence rates of melanoma continue to rise, with estimates that 106,110 new cases of invasive melanoma will be diagnosed in 2021 [3]. This is an increase of 62% from the 65,647 cases diagnosed in 2011 [4]. The rise in melanoma cases highlights the ever-increasing importance of the prevention and screening for this condition, which is especially important as the earlier detection of invasive melanoma improves the survival rates [5]. Further, it is likely that an increased awareness of melanoma risk factors and improvements in prevention methods, coupled with better treatments for advanced disease, will contribute to further declines in melanoma mortality.

Melanoma prevention and early detection are critical to limiting its morbidity and mortality in the United States. In this review, we assess the role of hereditary risk factors and environmental factors, principally sun-seeking behaviors, in melanoma and how these factors impact melanoma prevention strategies. In this context, we review the mixed evidence surrounding the use of sunscreen, community outreach programs, chemopreventive methods, and screening programs. We also address socioeconomic and cultural forces impacting melanoma prevention. Finally, we discuss the methods and techniques in melanoma imaging that are poised to improve the detection of this malignancy.

## 2. Heritable Etiologies for Melanoma Development

### 2.1. Genetic Syndromes That Predispose to Melanoma Development

Between 7 and 15% of patients with melanoma have a family history of the disease [6], and the risk of melanoma increases two-fold if a first-degree relative has been previously diagnosed with melanoma. The majority of the risk is likely related to shared sun exposure among family members, with fewer cases thought to be heritable in nature. While it is known that patients with genetic syndrome are at an increased risk of melanoma, differences in the features between these hereditary melanomas and sporadic melanomas have yet to be elucidated. Genetic conditions with high penetrance are discussed below.

The most common hereditary melanoma condition is familial atypical multiple mole melanoma (FAMMM) syndrome. These patients are at risk for cutaneous melanoma, pancreatic cancer, and neural system tumors, among other malignancies. Criteria for the diagnosis of FAMMM syndrome include a family history of cutaneous melanoma in a first- or second-degree relative; >50 total nevi with multiple atypical nevi; and specific concerning histologic features in nevi, such as asymmetry, subepidermal fibroplasia, and lentiginous melanocytic hyperplasia with spindle or epithelioid melanocytes, among others [7]. This syndrome is most commonly associated with a mutation in the CDKN2A gene, though it has also rarely been described with CDK4 mutations. In total, 45% of the melanomas tied to a genetic etiology are associated with a known germline mutation in CDKN2A or CDK4 [6]. CDKN2A is a gene located on chromosome 9p21.3 that codes for p16 and/or p14ARF, two inhibitory proteins. Both missense and nonsense mutations have been implicated in FAMMM syndrome. P16 is a cyclin-dependent kinase involved in the Rb (retinoblastoma) pathway, and a mutation results in dysregulated cell division due to uninhibited G1-S phase transition. Meanwhile, p14ARF regulates ubiquitination of the tumor suppressor p53, tagging it for degradation in the proteasome. The dysfunction of P14ARF impairs the DNA damage response [7,8].

BAP1 (BRCA1-associated protein 1) is another well-described gene associated with melanoma development. BAP1 is located on chromosome 3 and encodes a protein involved in the proteasome degradation pathway. Mutations in BAP1 are associated with cutaneous melanoma, uveal melanoma, and other internal malignancies such as mesothelioma and renal cell carcinoma [9]. These patients present with multiple, distinct melanocytic lesions that typically appear after the first few decades of life and usually vary between 5 and 50 in number. Another distinguishing feature is the presence of melanocytic BAP1-mutated atypical intradermal tumors (MBAITs), which are melanocytic lesions adjacent to tumors that stain negative for BAP1 [7]. Although they are benign, they can appear early in life and can thus be helpful in identifying patients for genetic testing. Interestingly, though the BAP1 protein associates with BRCA1, the data to support melanoma predisposition in carriers of either BRCA1 or BRCA 2 is mixed. Of the two, BRCA2 has traditionally been described with a stronger, albeit still relatively weak, link to melanoma development [10,11], though recent data indicates neither BRCA1 nor BRCA2 are likely to have a significant impact on the melanoma risk [12,13].

Other genes that have been studied for their association with melanoma syndromes include TERT, PTEN, and MITF, among others [7]. TERT, a reverse transcriptase, is one of the main components of telomerase, an enzyme that functions to elongate telomeres. Mutations in the promoter region of TERT have been associated with both germline and somatic cases of melanoma, as well as various other malignancies. Mutations in the POT1 gene—a component of the shelterin complex that acts to protect the telomeres—have also been shown to play a role in familial melanoma. PTEN hamartoma tumor syndrome (PHTS) is associated with germline mutations in the PTEN tumor suppressor gene. Of the four PHTS subdivisions, Cowden syndrome is most associated with malignancy and is a rare disease that presents with trichilemmomas, papillomatous papules, mucosal lesions, and palmar-plantar keratosis. In addition to its association with melanoma, this syndrome often presents with breast, endometrial, colorectal, and thyroid gland malignancies. Finally, Microphthalmia-associated transcription factor (MITF) functions to regulate the expression of a collection of genes that play an important role in melanocyte proliferation and invasion, and a substitution mutation in the MITF gene results in a fivefold greater risk of developing melanoma, renal cancer, or both [14]. For detailed descriptions of the molecular roles of TERT, POT1, PTEN, and MITF, we encourage readers to consult the recent reviews aimed at exploring the function and disfunction of each gene [15,16,17,18].

### 2.2. Heritable Traits Associated with Melanoma Risk

A number of nonmodifiable heritable traits increase one’s susceptibility to melanoma. For example, an increase in the number of either common or atypical nevi confer greater risk for malignancy, with the presence of just one atypical nevus associated with a relative risk (RR) of 1.60 [19]. The Fitzpatrick classification system assigns skin phototypes based on the response to sun exposure, such as a tendency of skin to burn versus tan. Differences in the response generally depend on both the skin’s melanin content and composition, with the darker eumelanin serving as a better photoprotector than the lighter pheomelanin [20]. A Fitzpatrick skin phototype (FST) of type I indicates skin that always burns and never tans in response to sun exposure, and each subsequent type denotes less burning and a better tanning response than the last. Having a type I FST conveys a RR of 2.09 compared to a type IV phenotype. Having lighter hair color also harbors increased risk, with red and blonde hair having an RR of 3.64 and 1.96, respectively, compared to dark-haired individuals. People with either blue (RR 1.47) or green (RR 1.61) eyes have a greater predisposition toward melanoma when compared to those with “dark” eyes. Further detail on the risk ratio associated with these heritable traits has been adapted from a series of excellent meta-analyses from Gandini et al. and are presented in Table 1 [19,21]. In addition, the increased risk of melanoma associated with these traits was further corroborated more recently by Olsen et al. 2019 in various updated models presented as part of the QSkin study [22].

Of note, genetic variants of the melanocortin 1 receptor MC1R are linked to high-risk phenotypic alterations in pigmentation—such as fairer skin, hair, and eye colors—that confer a predisposition to melanoma [23]. Aside from these phenotypic changes, recent studies also suggest that MC1R variants can independently elevate the risk of melanoma development [24].

### 2.3. Genetic Screening Recommendations

Given the increased risk for melanoma in people with the genetic alterations described above, genetic testing should be performed for indicated individuals [25]. The international guidelines developed in 2009 suggest patients be tested for CDKN2A based on the following: number of primary melanomas in the patient, melanomas in blood relatives, and pancreatic cancer in the patient or blood relatives [26]. The incidence of melanoma in a given region is also taken into account in these guidelines. More recent studies suggest updated scoring systems that incorporate the personal or family history of other malignancies that may be concerning for a familial syndrome. A personal or family history of cancers associated with melanoma—including prostate, breast, and colon cancer—also warrant further genetic workups [25]. In addition to proband testing, family members of patients who are offered genetic testing are also likely to get tested. Specifically in patients with CDKN2A mutations who saw a genetic counsellor, 98% of family members decided to undergo genetic testing, and 93% desired a referral for yearly skin examinations [27].

### 2.4. Monitoring of High-Risk Patients for Melanoma Development

A comprehensive screening strategy should be in place for patients with a known genetic predisposition for melanoma and for those diagnosed with FAMMM syndrome, especially because those with a CDK2NA mutation have a lower median age of onset of melanoma [28]. Patients with characteristics concerning for a familial syndrome should undergo full-body skin exams every six months supported by dermoscopy and total body photography. Sequential digital dermoscopy imaging (SDDI), a form of serial imaging that surveilles lesions of concern over short-term (3 months) or long-term (6–12 months) periods, can be used to help diagnose those at the highest risk. These guidelines have been shown to improve the early diagnosis in primary melanomas [29]. Using these techniques, detected melanomas are thinner than those found by traditional screening techniques [30,31]. While most studies conducted in order to make screening recommendations were performed only in dermatology clinics, newer data suggest that these surveillance programs can be expanded to additional practices such as primary care skin cancer clinics [32].

For specific rare genetic syndromes, formal surveillance recommendations are sparse. Skin examinations every six months should be conducted, and patients with a known BAP1 mutation should have MBAITs removed as well as other lesions that change with time, along with ophthalmologic screening every year to monitor for uveal melanomas [33,34]. In addition to more frequent clinical screening examinations, individuals in such high-risk groups should be carefully counseled on the importance of behavioral modifications to aid in melanoma prevention, including the proper use of sunscreen, use of protective clothing, and other UV avoidance measures (see “Risk of Sun Exposure” for further discussion).

At this time, genetic testing is largely reserved for the higher risk populations described above. Further elucidation of the genetics underlying hereditary melanoma development will be an important step towards the improvement of melanoma prevention practices.

## 3. Sun-Seeking Behaviors and Melanoma Development

### 3.1. Exposure to Sunlight Confers Risk of Melanoma Development

Ultraviolet A (UVA) and ultraviolet B (UVB) radiation are carcinogenic, and their role in melanoma development have previously been well-described in other literature [35,36]. Briefly, the mutagenic effects of UVA that are tied to melanoma development include the generation of DNA strand breaks, as well as reactive oxygen species that can indirectly damage DNA [37]. UVB is less abundant than UVA but can damage DNA at lower dosages, with its main effect on DNA involving the direct generation of cyclobutane pyrimidine dimers and pyrimidine (6–4) pyrimidone photoproducts [37]. Further, ultraviolet radiation (UVR) exposure is not homogenous and varies according to geographies and latitudes. The UV index (UVI) is a scale that increases proportionally to the intensity of erythema-causing UV doses on the Earth’s surface, with higher UVIs representing larger UVR dosages in a given timeframe. Latitudes closer to the equator tend to exhibit higher average UVIs than those closer to the poles, and geographical UVI has been demonstrated as a significant predictor of melanoma risk [38]. Overall, an extended exposure to UVR via sunlight or indoor tanning platforms is one of the most significant risk factors for developing cutaneous melanoma.

Casual sun exposure can be dangerous, particularly for high-risk individuals. This risk is present beginning in childhood, as sunburns in childhood are linked to an elevated risk of melanoma development [39]. Greater levels of UVR exposure also pose a risk to individuals who work in outdoor work environments, such as farmers, miners, and construction workers, as well as those living in locations with higher average UVIs. However, while it is recommended to limit UVR exposure when possible, it is not feasible to entirely avoid sunlight. Further, the health benefits of outdoor activities have been well-documented. Measures to avoid UVR are highly recommended when exposure to sunlight is necessary.

Accessories that act as physical barriers to UVR absorption, such as hats, sunglasses, long sleeves, and other sun-protective clothing, are important measures for melanoma prevention. Sun avoidance and the use of long sleeve clothing have both been associated with decreased odds of sunburns [40]. A recent analysis of whole-genome sequencing (WGS) datasets from uveal melanomas suggests a more prominent role of UVR as a driver in uveal melanoma pathogenesis, underscoring the importance of wearing sunglasses when exposed to sunlight [41]. The overlapping use of photoprotective accessories and shade-seeking while outdoors minimizes UVR exposure and thus reduces the risk of sunburn and subsequent melanoma development. A combination of these behavioral modifications with the proper use of viable chemopreventive agents such as sunscreen will be best-suited for the effective prevention of melanoma.

### 3.2. Tanning Correlation with Increased Melanoma Incidence

Tanning is a dangerous practice. The age at tanning initiation, as well as the duration of tanning behavior, modulate both the risk of developing melanoma and the average age of diagnosis. Prospective data taken from the Norwegian Women and Cancer study found that individuals that extensively partook in indoor tanning had a 32% higher chance of developing melanoma compared to never users [42]. Additionally, individuals who initiated tanning before the age of 30 had a 31% increased risk of melanoma. Tanning bed usage and the initiation of tanning behavior at a younger age have both been implicated in incidences of melanoma at a significantly younger age [42,43]. Beyond the risk of developing an initial melanoma, indoor tanning practices are significantly associated with the development of multiple primary melanomas [44]. Given the increased risk of melanoma in the context of tanning behavior, tanning is not a recommended practice for either vitamin D supplementation or for cosmetic reasons.

### 3.3. Tanning Impact on Melanoma Genotype

The impact of tanning is documented in the genomes of melanoma tumors. Excessive UVR exposure due to tanning behavior has been shown to affect the genetic characteristics of melanomas. The dosage of UVR per exposure and the chronicity of exposure both contribute to varying melanoma genotypes. The BRAF V600E genotype was found to be more prevalent in patients that engaged in indoor tanning compared with never users and was also shown to be more prevalent in patients that initiated tanning before the age of 25 compared to patients that began tanning later in life [45]. The next-generation sequencing of melanoma patient samples further demonstrated that 57% of the patients who frequented tanning salons harbored one of the BRAF mutations compared to 18% of the patients who had never used tanning salons [46]. Chronic exposure to sunlight in the context of an outdoor occupation was associated with a higher prevalence of BRAF V600E mutations as well [46]. Additionally, a history of blistering sunburns was linked to a 44% higher incidence of mutations in a collection of oncogenes, including a significant increase in the prevalence of P53 mutations and 75% more NRAS mutations. The contribution of tanning behavior to non-wildtype genotypes is of additional concern. NRAS^+^ melanoma genotypes are associated with lower degrees of lymphocyte infiltration, and BRAF^+^ genotypes are associated with a younger age of diagnosis. Both genotypes are associated with mitoses and are indicators of a poor prognosis for high-risk melanomas (AJCC T Stage > T2a) [47].

## 4. UV Addiction

### 4.1. Biological Basis of UV Addiction

UV addiction, also referred to as sun-seeking behavior, poses a potential challenge to the reduction of tanning behavior. It has been shown that individuals that frequently engage in tanning behavior demonstrate a strong dopamine-based neural reward response upon exposure to UVR, a response similar to that seen in many addictive disorders [48]. The production of β-endorphin in the skin upon exposure to UV has been implicated as a crucial actor in the addictive behavioral response. In mice, chronic UVR exposure is associated with elevated levels of β-endorphin in circulation. This elevation in serum β-endorphin occurred in conjunction with opioid-mediated behaviors. Upon the cessation of UVR exposure, β-endorphin levels returned to baseline and common opioid withdrawal symptoms were observed [49]. Readers are encouraged to examine recent reviews for more in-depth discussions of the physiological pathways of UV addiction and the general biological effects of UVR [50,51].

### 4.2. Genetic Predisposition toward UV Addiction

The evidence of addictive behavior linked to UV is important in the context of melanoma prevention. Consideration should be given to identifying risks for the development of sun-seeking behavior. Recently, the possibility of a genetic predisposition to the behavior has been explored. A study comparing the behavior of monozygotic and dizygotic twins supported the conclusion that sun-seeking traits have a considerable genetic component [52]. The researchers continued by performing a genome-wide association study (GWAS) in large samples from the U.S. and United Kingdom and found that the variants in five genetic loci—including MIR2113, CADM2, and TMEM182—are closely associated with sun-seeking behavior. Interestingly, the loci identified have been previously linked to addictive behavioral traits [53,54,55].

### 4.3. Role of Vitamin D in UV Addiction

The role of vitamin D in the development and modulation of sun-seeking behavior has also been recently highlighted. The reduction of vitamin D levels has been suggested to sensitize individuals to the β-endorphin and endogenous opioid reward pathways, thereby causing a feedback loop whereby UV exposure is rewarded to maximize vitamin D synthesis [56]. This pathway involving vitamin D has important implications for reducing UV exposure and sun-seeking behavior. Firstly, vitamin D supplementation can be emphasized as an effective and safe alternative to UV exposure for the daily vitamin D intake, thereby reducing the likelihood of developing UV dependence within the population. More conclusive research is needed, but the recent data suggest that vitamin D may decrease the sensitivity of the UV/β-endorphin axis and, thus, may be helpful in reducing the sun-seeking behavior of individuals with UV addiction. Overall, vitamin D supplementation could help mitigate a difficult challenge in decreasing UV exposure in an effort to reduce melanoma incidence.

### 4.4. Declining Tanning Prevalence in the United States and Australia

Given the risks associated with tanning behavior, the reduction of tanning prevalence is a major public health concern. The Health Information National Trends Survey (HINTS) indicated that 10% of surveyed adults in the United States engaged in indoor tanning in 2007 [57]. The prevalence of indoor tanning has steadily decreased since that time, and the updated HINTS report indicated that 4% of U.S. adults engaged in indoor tanning in 2018 [57,58]. This declining trend in indoor tanning was also found among individuals under the age of 18 in the United States, with the national Youth Risk Behavior Survey reporting a decrease in prevalence from 15.6% in 2009 to 7.3% in 2015 [59]. The decrease in indoor tanning practices among both adults and minors within the U.S. is promising, especially since indoor tanning is specifically associated with an increase in sun burns [58,59].

In Australia, the national Slip! Slop! Slap! campaign, which debuted in 1980 and evolved into the currently operational SunSmart campaign in 1988, attempted to increase nationwide awareness of the dangers of UV exposure and tanning behavior. This program helped to significantly shift the public attitude related to sun exposure and tanning. For example, the percentage of Australians desiring a tan fell from 61% to 35% between 1988 and 1998, and the percent agreeing that the summer is more easily enjoyed after a tan decreased from 62% to 29% [60]. In recent years, the Australian government has also taken legal measures to reduce the prevalence of indoor tanning practices. Australia progressively restricted indoor tanning access, beginning with the prohibition of tanning for minors, until 2016, when the government instituted a population-wide ban on the use of commercial solaria. This ban is projected to prevent the development of 31,009 melanomas and avert 3017 melanoma deaths in a cohort of 6.95 million young Australians, and the legislation restricting solaria usage also appears to have been linked to decreased consumer interest in UV-based indoor tanning, as well as increased interest in fake/spray tanning alternatives [61]. Strategies similar to those employed in Australia, such as organized educational campaigns regarding the carcinogenic nature and risks of tanning and/or restrictions on indoor tanning device usage, can be considered to further decrease tanning activity in the United States.

## 5. Therapeutic Approaches for Primary and Secondary Chemoprevention: Current Clinical Evidence

The primary chemoprevention of cutaneous melanoma refers to the use of pharmaceutical interventions to reduce the likelihood of oncogenesis in healthy or sun-damaged skin. Secondary chemoprevention encompasses therapies that prevent premalignant entities, such as dysplastic nevi, from converting to melanoma. While numerous agents have shown promise through in vitro and animal studies, few have made the successful transition to trials in human subjects. Multiple excellent reviews have summarized prior in vitro and in vivo animal studies of potential chemopreventive agents, including Jeter et al. 2019, Chhabra et al. 2017, and Mounessa et al. 2016 [62,63,64]. The discussion in this section will therefore be limited to the current chemoprevention agents with clinical data. Interventional studies demonstrating a reduction in the incidence of cutaneous melanoma as their primary endpoint represent the highest level of evidence. However, due to the long observation periods required to document such a change, many studies do not set this as a primary endpoint. Therefore, studies investigating the effect of chemoprevention on biomarkers of cutaneous inflammation, as well as those measuring the incidence of keratinocyte carcinomas, which share a common UV-mediated diathesis with cutaneous melanomas, are reported in this section.

### 5.1. Sunscreen

#### 5.1.1. Types of Sunscreens

In addition to the use of protective clothing and glasses, the use of sunscreen is paramount and highly recommended by dermatology and cancer organizations internationally [65,66,67]. Among 540 polled practicing dermatologists in the U.S., 99% recommended the regular use of sunscreen [68]. Sunscreens function by raising the minimal erythema dose (MED) of UV light needed to induce cutaneous inflammation with resultant erythema. Sunscreens are typically divided into two classes based on their active, UV-protective ingredients: organic and inorganic. Common organic UV filters include avobenzone, octinoxate, octocrylene, oxybenzone, and padimate O and function by converting UV energy into heat. Recently, there has been concern raised regarding the potential systemic absorption of organic ingredients, leading to possible endocrine disruption. Inorganic UV filters, including micronized zinc oxide and titanium dioxide, function by blocking UV light directly [69]. Systemic absorption is not a concern for these sunscreens. However, inorganic products typically leave an opaque, white cast on the skin due to their large particle size, which can be a cosmetic limitation to their daily use for certain patients. A recent study investigated the use of padimate O nanoparticles in a topical sunscreen formulation. These particles were engineered to be bioadhesive to reduce the risk of systemic absorption, and they achieved noninferiority to the commercial standards in murine models [70]. A phase I clinical trial of this nanoparticle-based formulation was completed in 2017, with the results still pending publication.

#### 5.1.2. Data Addressing the Use of Sunscreen

Sunscreen use is nearly universally recommended for the prevention of melanoma and other adverse UVR-related skin effects, with estimates that a 5% increase in sunscreen usage in the U.S. population per year over a 10-year period could prevent upwards of 231,053 melanomas [71]. Analyses of the efficacy of sunscreen, however, have yielded mixed results. For example, though the only randomized controlled trial to assess the effect of sunscreen usage on melanoma incidence indicated a reduction of melanoma in the sunscreen usage group [72], it should be noted that the results did not reach the traditional levels of statistical significance. Follow-up studies of differing designs have been conducted, such as a population-based cohort study that correlated the use of SPF ≥ 15 sunscreen with decreased melanoma incidence [73]. Another case-controlled study in Australia found that sunscreen use during childhood, as well as lifetime sunscreen use, indicated a lower risk of developing melanoma [74]. However, a meta-analysis of the studies consisting of case–control, two-cohort, cross-sectional, and controlled trial designs did not find an overall significant association between the sunscreen usage and melanoma development [75]. More recently, another meta-analysis revisited the association between sunscreen and melanoma and noted a remarkable heterogeneity across case–control studies and study designs [76]. The adjustment for common confounders—including sun exposure and sun burns—systematically shifted the results toward a reduced risk of melanoma with sunscreen usage, though the study concluded that the evidence from the observational studies was heterogeneous and weak.

The results from this study also provided important insights into the data regarding sunscreen use, especially regarding potential confounders to consider when assessing sunscreen effectiveness. For example, the usage of sunscreen has been connected to an increased duration of intentional sun exposure [77]. Additionally, differing techniques of sunscreen application may also contribute to the lack of efficacy observed in noncontrolled study designs. A linear relationship between the time spent applying sunscreen and photoprotective body coverage exists [78], indicating that cursory applications of sunscreen might not convey the intended level of UV protection. Recent studies have also indicated that typical consumer sunscreen application routines neither provide complete body coverage nor reach the recommend sunscreen area density [79,80]. Education campaigns to improve sunscreen application practices and sunlight avoidance, even among regular sunscreen users, should be considered to maximize any protective effect of sunscreen usage in the population.

Understanding consumer preferences and attitudes toward sunscreen could help improve sunscreen usage and application practices. Sharma et al. 2020 found within a cohort of skin cancer patients that individual sunscreen use patterns were linked to the perceived magnitude of melanoma risk reduction due to sunscreen use [81]. A study assessing tanning habits among German participants found that the avoidance of solar dermatitis was cited as a greater motivation for sunscreen use than the prevention of skin cancer [82]. The study also identified that skin aging was considered an especially important motivating factor among female respondents, and the laboriousness of sunscreen usage was commonly cited as a reason for an opposition toward applying sunscreen more frequently among men. Educational campaigns that target individual attitudes toward sunscreen and motivations for and against its usage may be helpful in improving sunscreen practices among the population.

#### 5.1.3. Novel Methods to Improve Sunscreen Usage and Efficacy

Efforts are currently underway to develop novel methods of improving sunscreen use. One such approach is centered on the idea of sunscreen smart stations that can be placed near outdoor workplaces or areas where extended UVR exposure is common [83]. These smart stations would be intended to increase the accessibility to sunscreen and facilitate the ease of reapplication. Another approach is the use of wearable UV sensors for the monitoring of UVR exposure in users. The use of UV detection stickers has been shown to dramatically increase sunscreen usage among wearers both in clinical trials and real-world settings [84,85].

Other high-tech monitoring systems are also under development. One example involved the use of UV sensors in conjunction with a smartphone to provide real-time feedback regarding UV dosage. These sensors have been shown to modulate wearers’ behavior in regard to UV exposure, with a resulting decrease in the average UV dosage received per day [86,87]. Application-based monitoring systems could also prove to be efficacious for increasing sunscreen usage, especially among individuals at high risk for melanoma. Overall, increasing sunscreen access via sunscreen smart stations, as well as UV exposure tracking, are promising methods for improving sunscreen practices in the population. In conjunction with the educational campaigns discussed previously, these methods may also lead to a decrease in melanoma incidence.

### 5.2. Non-Sunscreen Chemopreventive Agents

#### 5.2.1. Nicotinamide

Nicotinamide, or niacinamide, is the amide form of vitamin B3 and has shown significant antioxidant, antiproliferative, and anti-immunosuppressive effects in preclinical studies of skin exposed to UV radiation. Patients in the ONTRAC (Oral Nicotinamide To Reduce Actinic Cancer) study who received nicotinamide experienced 23% fewer keratinocytic carcinomas and 13% fewer actinic keratoses than the placebo group, but no significant differences were noted in the incidence of melanomas in the study group [88]. Of note, the study was underpowered for the melanoma endpoint comparison, as only ten melanomas were diagnosed in the study participants compared to 801 nonmelanoma skin cancers. No current trials are further investigating nicotinamide as a chemopreventive agent. A phase I trial studying topical myristyl nicotinate (MN), a topical niacin derivative, for chemoprevention in healthy volunteers was completed in 2016 but did not meet its primary endpoint of safety versus a placebo, with 36.36% of on-treatment patients experiencing adverse cutaneous events versus 9.09% in the placebo arm [89].

#### 5.2.2. Nonsteroidal Anti-Inflammatory Drugs (NSAIDs)

Enhanced prostaglandin synthesis—with COX-2 overexpression, in particular [90]—may be involved in the oncogenesis of cutaneous malignancies. This theory has led to the study of NSAIDs as chemopreventive agents, though meta-analyses of epidemiologic studies have not proven conclusive. Most meta-analyses suggest no effect of long-term NSAID use on the incidence of melanoma. A slightly decreased risk of melanoma was noted in aspirin users in three analyses of case–control studies, with one analysis suggesting no effect of aspirin on melanoma incidence [91,92,93]. Despite mixed results from these studies, numerous clinical trials have investigated the use of NSAIDs as cutaneous chemoprevention agents.

The most promising data for NSAID use was suggested in studies of celecoxib for the prevention of keratinocyte carcinomas. In one trial of 240 subjects with 10–40 actinic keratoses who received celecoxib vs. a placebo, there was a 43% lower risk of developing nonmelanoma skin cancer in the patients treated with celecoxib versus a placebo, and the patients tolerated treatment without adverse events. No difference was noted in the incidence of actinic keratoses between the two groups [94]. Another double-blind, placebo-controlled, randomized phase II study investigated the chemopreventive efficacy of oral celecoxib, 200 mg twice daily for 24 months, on the number of basal cell carcinomas (BCCs) in patients with basal cell nevus syndrome. Celecoxib decreased the number of new BCCs and BCC burdens in patients with <15 BCCs at baseline but did not have a significant effect on subjects with ≥15 BCCs at baseline [95].

A phase II, randomized, double-blind, placebo-controlled trial of oral sulindac versus a placebo for eight weeks was conducted in 50 individuals with atypical nevi. Sulindac metabolites were found to be sufficiently delivered to the skin and plasma, though only a trend toward an increased expression of the apoptotic marker that cleaved caspase-3 was noted in atypical nevi when adjusted for the baseline expression [96].

A topical formulation of diclofenac with or without difluoromethylornithine (DFMO) was studied in a Phase IIB trial in 156 patients for the prevention of keratinocyte carcinomas. The subjects were randomized to either topical DFMO twice daily, topical diclofenac daily, or DFMO plus diclofenac, with the primary measured outcome as a karyometric average nuclear abnormality (ANA) in the treated skin. The ANA was increased across all groups, suggesting the topical agents actually increased cutaneous inflammation [97].

Due to drug-specific effects noted in case–control and cohort studies [91,92,93], aspirin is currently being investigated as a chemopreventive agent in two registered trials. The preliminary results from an open-label, nonrandomized study of 41 subjects receiving either 325-mg or 81-mg aspirin daily for one week revealed the detection of salicylate in every nevus sample following aspirin use. Higher salicylate levels were detected from the subjects in the 325-mg cohort. Functionally, the PGE_2_ levels were suppressed in nevi, with a reduction of 50–70% and 35–50% for the 325-mg and 81-mg cohorts, respectively [98]. This data suggests further research is indicated, and a placebo-controlled phase II trial is currently underway.

Of note, numerous NSAIDs have been associated with phototoxic and/or photoallergic reactions [99]. Though the use of phototoxic medications has been suggested as a potential etiology behind skin cancer development [100], the evidence surrounding a linkage between NSAID use and skin cancer has been mixed to date [101]. A recent study analyzing the use of NSAIDs specifically derived from propionic acid—including naproxen, ketoprofen, and ibuprofen—has suggested an increased association with cutaneous melanoma development [102].

Though several of the NSAIDs presented here, including diclofenac and celecoxib, have been linked to phototoxic skin reactions [103,104,105], these agents are not propionic acid derivatives. Even so, the continued consideration of this association would be a prudent measure before any potential initiation of an NSAID as a chemopreventive measure. Readers with further interest in the relationship between photosensitizing medications and skin cancer are encouraged to explore George et al.’s recent and excellent in-depth discussion of this topic [106].

#### 5.2.3. Vitamin A Derivatives

Vitamin A derivatives function by binding to the retinoic acid receptors (RARs) alpha, beta, and gamma, along with retinoid X receptors (RXRs). While the mechanism of action behind vitamin A derivative-induced chemoprevention has not been clearly elucidated, it is suggested these agents may have anti-inflammatory and antiproliferative effects beneficial to preventing cancer growth. However, both oral and topical agents have failed to demonstrate clear efficacy in clinical trials.

In a study of five patients with xeroderma pigmentosum treated with daily oral isotretinoin for two years, there was an average reduction in new skin cancers of 63%. When isotretinoin was discontinued and the patients were followed for an additional year, the rate of new tumors increased to an average 8.5-fold higher number than the frequency observed during treatment [107]. However, in another randomized, double-blinded, placebo-controlled trial of 525 participants with a history of at least four keratinocyte carcinomas randomized to receive oral retinol, isotretinoin, or a placebo supplementation daily for three years, no difference in keratinocyte cancer incidence was observed between either experimental arm compared to the placebo [108]. In a study of oral acitretin, 70 patients with a history of at least two keratinocyte carcinomas within five years of the trial onset were randomized to either acitretin 25 mg orally five days per week or a placebo, and no statistically significant reduction in the rate of new primary keratinocyte carcinomas was found after two years [109]. Finally, in a robust phase III, placebo-controlled trial of 1131 patients randomized to either topical tretinoin 0.1% twice daily or a placebo for 1.5–5.5 years, no significant difference in keratinocyte carcinoma incidence was noted in the tretinoin arm. An additional study was registered investigating topical tretinoin with or without oral fenretinide in patients with dysplastic nevus syndrome, but the results from this study remain unavailable [110].

#### 5.2.4. N-Acetylcysteine (NAC)

N-acetylcysteine has antioxidant properties in vivo and has therefore been investigated as a chemopreventive agent for melanoma. In one phase II, double-blind placebo-controlled study, 100 patients were randomized to either a single dose of NAC or a placebo. A solar radiation simulator was used to irradiate one nevus on each subject with one to two MED, and both the irradiated nevus and a control were taken for analysis of the biomarkers related to UV-induced oxidative stress and NAC metabolism on the prior day. Importantly, no significant difference was noted in the markers of UV-induced oxidative stress between the treatment and placebo groups [111]. In addition, in an open-label post-trial study of ten patients in which NAC was taken three hours before nevus removal, similar nonsignificant results were observed.

#### 5.2.5. Vitamin D

In a double-blinded, placebo-controlled trial, 20 subjects were randomized to receive vitamin D_3_ or a placebo one hour after an experimental MED sunburn. In the skin biopsy specimens collected 48 h after a sunburn, the patients in the vitamin D-treated arm had significantly lower levels of proinflammatory mediators TNF-α and iNOS. Moreover, the patients with higher serum vitamin D_3_ levels after treatment demonstrated an increased expression of anti-inflammatory mediator arginase-1 and a sustained reduction in erythema [112].

A randomized, placebo-controlled trial investigating the effect of oral vitamin D_3_ supplementation on melanocyte biomarkers in 24 patients was completed, with over 270 genes differentially expressed in melanoma versus benign nevi showing changes in expression after the vitamin D treatment. A complete analysis of the data has yet to be published.

A summary of the efficacy of these and other potential agents—including statins, T4 endonucleases, DFMO, and dietary agents/vitamins/minerals—with the clinical results (if available) presented in Table 2.

The evidence surrounding the use of chemopreventive therapeutics for melanoma prevention is varied, and further research is increasingly needed to determine the true clinical efficacy of these agents.

## 6. Socioeconomic Risk Factors for Melanoma Development

### 6.1. Factors Influencing Public Awareness

Previous literature has suggested that education, awareness, and primary prevention of melanoma vary due to factors such as race, sex, socioeconomic status (SES), family history, and age [113,114,115]. For instance, older-aged individuals and females tend to seek medical advice and utilize self-examination most frequently, and professionals or those with higher education are more likely than the average population to receive specialist care [115]. With regards to race, White individuals are more likely to be knowledgeable about the benefits of skin surveillance measures compared to White Hispanic and non-Hispanic Black individuals [113,115,116]. A perceived risk is associated with awareness and is the result of race, SES, family history, and age [113,115,116,117]. These inherent and circumstantial factors suggest that melanoma education should be specific to community demographics for the maximum effect [113,114,117].

The decreased public perception of melanoma risk in darker skin types is a barrier to melanoma prevention efforts [113,115,116]. One survey found that, for individuals with FST I and II, 86% knew melanoma was a type of cancer, compared to 46.3% and 57.6% of FST III/IV and FST V/VI individuals, respectively [113]. This may be attributed to a reduced likelihood of familial or personal history in groups with a lower incidence of melanoma, as the resultant anecdotal knowledge of skin cancer increases the perceived risk and encourages individuals to perform and receive skin examinations [118]. The primary prevention methods of melanoma such as self-skin examinations or dermatologist consultations may be underutilized in communities that underestimate their risk. It is therefore important to educate individuals of all skin types about melanoma to increase the implementation of primary prevention measures.

### 6.2. Current Outreach Programs to Address Cultural and Community Barriers to Prevention Efforts

Currently, there are multiple organizations advocating for education about melanoma and nonmelanoma skin cancer. The United States Preventive Services Task Force (USPSTF) in 2018 recommended counseling different demographic groups about skin cancer prevention [119]. These guidelines describe a moderate benefit in behavioral counseling of fairer-skinned individuals and encourage physician-mediated education about skin protection and self-skin examinations [119]. However, the USPSTF acknowledged that counseling can only be recommended to fair-skinned individuals due to the lack of research regarding skin cancer counseling in darker-skinned people [119]. The further inclusion of more varied skin colors in skin cancer research can determine if people with darker skin could benefit from similar behavioral counseling. Since 2002, the United States Center for Disease Control (CDC) has upheld guidelines for school programs to provide skin cancer education to children, but they are not strictly followed [120]. Further, the CDC school program guidelines are generic across schools and do not consider cultural factors that affect health literacy, such as variations in the SES among different schooling districts. Cultural barriers such as language and social relevance should be addressed to increase the accessibility and success of melanoma-related school education [113,117].

Projects addressing cultural diversity and relevance have shown promise in educating otherwise overlooked communities. Providing melanoma educational materials in different languages is one way to improve community understanding of the condition. One study found that an online skin cancer video offered in Spanish to Hispanic communities increased their knowledge about melanoma and the incorporation of skin self-examinations in practice [121]. Though these online curriculums aid those with online access and prove useful in raising melanoma awareness in these communities, these programs face some limitations in areas with low SES due to a decreased access to smartphones and/or other required technologies.

The rise of social media and widespread accessibility to social media platforms provide ample opportunity for educating the public. Skin cancer awareness campaigns have shown a strong correlation with increased online information-seeking; these campaigns have been shown to lack diversity and overlook people of color [122,123]. One study of these campaigns revealed 100% of posted skin cancer lesions and 72.9% of posts targeting at-risk groups were the FST I/II skin type, while the skin cancer risk in people of color was referenced textually 1.7% of the time [123]. Underrepresentation is a major limitation of otherwise effective melanoma awareness campaigns in the media.

### 6.3. Access and Barriers to Specialist Care

#### 6.3.1. Disparate Access to Skin Exams

Access to medical professionals is imperative to melanoma prevention. Although skin self-examinations are practical and effective in detecting skin cancer, physician-mediated examinations are crucial for detecting thinner melanoma lesions resulting in increased melanoma-specific survival [124]. Skin examinations are disproportionately underperformed in communities of color, as one study found only 5% of minorities have received a total body skin examination by a physician in comparison to 49% of white individuals [117]. Despite the higher melanoma incidence in fairer skin types, darker-skinned individuals diagnosed with melanoma experience higher rates of morbidity and mortality related to melanoma [113,114,117,118]. The data from the Surveillance, Epidemiology, and End Results (SEER) Program in 2013 revealed the five-year melanoma-specific survival of Black individuals to be 67.3% compared to 91.1% for White individuals [125]. Specialist accessibility is positively correlated with increased preventive measures and early diagnosis; therefore, inconsistent accessibility poses an area for robust improvement [124].

#### 6.3.2. Insurance

Insurance coverage has been associated with reduced melanoma diagnosis higher than stage T1b in comparison to noninsured patients [126]. Studies found that expanded public insurance coverage under the Affordable Care Act’s Medicaid program has been correlated with a decrease in late-stage cancer diagnosis through increased access to medical professionals and increased affordability of care [126,127]. Though this is encouraging, other studies have found publicly insured patients to have, on average, thicker melanoma than privately insured patients, likely because public insurance is not universally accepted and can lead to less timely care [128].

#### 6.3.3. Geographic Barriers to Care

Areas with a higher density of dermatologists are correlated with the earlier diagnosis of melanoma [128,129,130]. One spatial analysis study found that a low density of clinics corresponded to a 30% increase in the risk for thick lesions [128]. The distance to dermatologist clinics increased the risk of thick tumors for White Hispanic individuals and poorer communities, which highlights the issue of a lack of accessibility to treatment due to racial and socioeconomic disparities [128]. Another study found that having access to 0.001-1 dermatologists per 100,000 people reduced melanoma mortality by 35%, and having access to 1.001-2 dermatologists per 100,000 people reduced melanoma mortality by 53% [129]. A spatial distribution analysis of specialists can identify vulnerable areas for non-specialist intervention through community outreach and nontraditional physician access, such as telemedicine programs.

#### 6.3.4. Telemedicine/Virtual Intervention

Due to the COVID-19 pandemic, telehealth programs have expanded and provided a glimpse into the high utility of virtual care. The expansion of telemedicine beyond COVID-19 can extend legitimate and convenient medical care to patients, as virtual health consultations may help alleviate the gaps in specialist care caused by some of the geographic and socioeconomic disparities discussed above. Teledermatology has increased access to providers by decreasing wait times and removing obstacles that interfere with care access, such as geographic location, scheduling, and travel [131].

Quality assurance is a crucial concern for the widespread implementation of teledermatology. Research shows a moderate-to-similar accuracy of virtual dermatology consultation compared to an in-person melanoma diagnosis [131,132,133]. However, the accuracy of teledermatology and skin cancer diagnosis varies across studies, demonstrating up to 90.91% accordance with a histopathological diagnosis [132,134]. The addition of teledermoscopy, the use of high-quality skin photographs, to telemedical consultations appears to facilitate the diagnosis of melanoma. One study found 100% sensitivity and 85% specificity of teledermoscopy for diagnosis, while another found an increase in specificity from 86.57% to 92.86% and sensitivity from 72.33% to 96.24% upon the addition of teledermoscopy [133,135]. Most studies agree that teledermoscopy increases the specificity of a remote malignancy diagnosis [133,134,135], and this validation may help to increase teledermatology utilization in those who cannot access specialists due to geographic, scheduling, and expense limitations. Barriers to teledermatology care, such as a disproportionate access to technology and insurance access issues for lower SES populations, remain. As these issues are addressed, telemedicine will likely grow in use and utility into the future for melanoma detection and management.

Other technological programs have been trialed for the at-home detection of melanoma lesions that do not require physician intervention and instead rely on artificial intelligence and smartphone applications [136]. Three melanoma smartphone applications were tested and found to have variable diagnostic sensitivity and specificity, with values as low as 21% and 27%, respectively [137]. While the results are mixed on the utility of these technologies, most of the applications analyzed were developed around 2013 and, therefore, do not reflect the same technological ability of today. Notably, earlier this year, Google previewed its “Derm Assist” program powered by artificial intelligence that aims to identify skin conditions through the analysis of images taken through a smartphone camera. Though promising in theory, the real-world efficacy of this program remains to be seen.

At-home applications that do not require a physician appointment have the potential to one day augment other melanoma prevention methods in communities that lack access to medical care, though these programs need much further refinement before any become truly viable for clinical diagnostic purposes. The further technological development in algorithms and cameras can improve accuracy and provide a useful and accessible tool for the primary prevention and early detection of melanoma [136,138].

#### 6.3.5. Non-Specialist Intervention

The virtual dermatologic education of family practitioners can increase the accessibility of physician examinations of melanoma. The teledermoscopy training of primary care providers (PCPs) is possible to further the expansion of non-specialist dermatologic care [139]. Primary care provider dermoscopy training has shown promise in under-resourced clinics by reducing the wait time for general care and providing access to dermatologic information and care plans [140]. The teledermoscopy training of PCPs increases the sensitivity and specificity of skin neoplasm identification [141]. One study demonstrated that trained PCPs referred significantly more melanomas and fewer benign lesions to specialists when compared to those that did not receive mastery training [142]. Though the virtual dermatologic training of non-specialists should not replace dermatologist intervention, these resources can prove valuable for improving patient care in presently under-resourced areas.

## 7. Screening and Image-Based Approaches to Secondary Prevention

The secondary prevention of melanoma is a critical area of research. Noninvasive screening examinations have been the de facto screening methodology, given the accessibility of skin for growth visualization, but the 2016 USPSTF guidelines did not recommend public screening programs for skin cancer, as insufficient evidence was cited for the efficacy of such programs [143]. Continuing research into the secondary screening of melanoma is especially of interest, because it can be greatly beneficial to survival, as early-stage melanoma detection is associated with a lower mortality [144]. The specific impact of the public implementation of secondary prevention on the mortality rates, costs of treatments, and overall overdiagnosis are areas that urgently need further study [145]. Screening is currently recommended by many for high-risk populations, where studies in recruitment methods, self-monitoring, and public awareness are underway [146,147,148].

An unaided total body skin examination is currently the most common screening method. The method uses the “ABCDE” methodology: asymmetry, border irregularity, nonuniform color, diameter greater than 6 mm, and evolution over time [149]. A physician-performed total body skin examination has been linked with a decreased incidence of thick melanomas, which are typically associated with poorer prognoses.

Screening methodologies for high-risk populations are not universally established. The prevalence of skin self-examinations remains low in high-risk groups [150], even though encouraged by the American Cancer Society. Full-body screening is possible but not widely implemented. Further, biomarkers and artificial intelligence are recently developed tools that have the potential to improve the accuracy of skin cancer screening.

Strategies for secondary prevention have been implemented and studied in other countries. In Australia, a targeted prevention and early detection program called SunSmart began in 1988 to reduce skin cancer incidence, morbidity, and mortality. Promoting both public awareness and screening practices significantly reduced the rates of melanoma [145]. In another endeavor in Schleswig-Holstein, Germany, the melanoma mortality rates were reduced by 47% and 49% in men and women, respectively, in the five years following the launch of a total body skin examination program [151]. In the absence of a national screening program in the United States of America, the University of Pittsburgh also investigated annual full-body exams for early melanoma detection via primary care physicians, which resulted in earlier diagnoses with 50% thinner melanomas [152]. These results suggest the value in implementing national screening programs and guidelines.

### 7.1. Imaging Techniques

New screening methods with higher sensitivities are also under development. Total body photography (TBP) images the entire skin surface at a single time point as a reference for longitudinal monitoring [149]. TBP typically is performed in imaging booths and captured within minutes. There exist both 2D and 3D methods of TBP. Two-dimensional TBP takes images of the skin and maps these images onto a human digital model. Three-dimensional TBP makes a representation of the patient without model assistance and with potentially higher locational accuracy, though the machinery is often more expensive and difficult to incorporate due to its size. Both methods of TBP offer increased accuracy in the tracking of nevi for melanoma development. The comprehensive nature of TBP makes this form best designed to detect lesions that arise de novo, and the current use of TBPs is mainly for patients with too many nevi to track individually. A recent review of 14 studies published between 1997 and 2020 found that the use of TBP can improve the early detection of melanoma in high-risk populations [153].

Temporal image comparison, or serial imaging, uses digital imaging over time to track the growth of potential cutaneous melanoma to assess their risk. SDDI is one method that is most useful for pre-existing lesions, as, by nature, it tracks them over time [149]. This method is especially useful for tracking lesions without clear benign or malignant features. In a prospective study of 212 high-risk patients, SSDI alone was used to diagnose 15 out of 17 melanomas, and further studies showed a 3.3-fold reduction in unnecessary biopsy procedures for diagnosis with SDDI. In addition to the demonstrated utility of SDDI alone, a combination of SDDI and TBP has also been shown to increase the diagnostic advantages [154].

Further notable and developing techniques for melanoma detection include reflectance confocal microscopy, optical coherence tomography, multiphoton imaging, and stepwise two-photon excited fluorescence. These are fairly new technologies with limited commercial availability and are discussed below.

Reflectance confocal microscopy (RCM) captures images of the cellular resolution of each skin layer in a stepwise fashion. These stacks are stored electronically and can be used for longitudinal study. RCM also uses the refractive index of melanin to help diagnose melanoma. In several studies from 2008 to the present, RCM has been shown to improve the sensitivity, specificity, and the detection rate of melanoma [155]. However, RCM is currently quite expensive, and the detection area and depth are limited [156].

Optical coherence tomography (OCT) utilizes the direct reflection of light as it passes through structures of varying optical densities. By measuring the difference of a reference laser beam and the reflected beam, an image can be constructed. OCT offers the added benefit of tracking progressions of lesions via alterations in vessel morphology. The application of OCT to melanoma screening remains in the early stages of development, as some studies have noted that the levels of sensitivity and specificity are not currently sufficient for acceptable use in diagnosis. Further investigation to improve the melanoma diagnosis quality of OCT may prove to be fruitful [157].

Multiphoton imaging (MPM) is able to image skin to a depth of 200 µm with sub-cellular resolution and can provide functional and structural information on unstained lesions. Combined with fluorescence, MPM can be quite site-specific, penetrative, and descriptive. This technique is also very new and costly, and artifacts from patient movement remain an obstacle to the further widespread adoption of MPM.

One-photon excitation is generally insufficient to detect the autofluorescence of melanin. A method to overcome the limitations of traditional one-photon fluorescence in tissue is known as dermatofluoroscopy, where a stepwise excitation of two photons results in fluorescence. This method is able to specifically detect melanin fluorescence without interference from background autofluorescence. As a diagnostic method, dermatofluoroscopy machines have been made commercially available [158], but further research is ongoing to develop the method.

### 7.2. Application of Artificial Intelligence to Melanoma Screening

Imaging-based skin cancer screening is naturally a suitable application for artificial intelligence (AI). While AI is not currently used clinically for prognostic efforts, work is being done to assess its potential. Machine learning (ML), a subset of AI, has been used to assess skin cancer, and ML’s ability to do so has advanced with the technologic advances in the field [159]. Trained AI algorithms have also shown the potential to outperform board-certified dermatologists in identifying images of malignant lesions [160,161], though melanomas originating from initially benign lesions are still unable to be predicted by clinicians and algorithms alike [162].

The accuracy and applicability of AI to melanoma screening, while promising, also needs further development and research. A review of 51 articles suggested that dermatologist involvement in artificial intelligence system building for skin cancer assessments is low, making the currently available systems of AI suboptimal, as they do not take advantage of dermatologists’ clinical expertise [159]. A qualitative study investigating patient attitudes towards AI use in skin cancer screening found that patients experienced increased anxiety and feared a less accurate diagnosis by AI systems, though patients were receptive to the use of AI that also maintained human physician–patient relationships [163]. Further, the AI in this setting may presently be lacking in skin-of-color lesion training, causing underperformances for patients of color [164]. The use of artificial intelligence for skin screenings is continuing to grow in robustness and clinical applicability and may soon become a key aid to dermatologists for the clinical management of cutaneous melanoma.

## 8. Conclusions

The effective prevention of melanoma is critical to the continued decline in melanoma mortality in the United States, and a multifaceted approach is indicated. Sociologic and community-based interventions, such as educational and community outreach programs that increase the awareness of melanoma, as well as proper sunscreen application, have demonstrated efficacy in this area. Similar interventions have also been associated with tanning behavior reduction, as seen in Australia following the implementation of the effective SunSmart awareness campaign. Though the United States does not presently have nationwide screening protocols, examples of successful programs in other countries provide a template for another effective method to decrease melanoma rates. Recent and further elucidation of the biological basis behind sun-seeking behavior may also help guide the development of public health measures to facilitate a decrease in tanning. In the meantime, the regular screening of individuals with elevated melanoma risk due to genetic and hereditary factors should be employed at intervals usually no greater than one year apart and often more frequently, depending on the specific indication. Chemoprevention is another active field of study that may help reduce the development of skin cancer. Few potential chemopreventive agents presently show demonstrable efficacy, with the current majority of promises also limited to keratinocytic carcinoma prevention as opposed to the prevention of melanoma. Finally, in addition to increased access to trained and qualified providers, the recent emergence of teledermatology as a viable clinical platform has presented as a ripe opportunity to allow for a greater reach of effective monitoring and care for dermatological malignancies. Further research into imaging techniques and artificial intelligence is underway and will undoubtedly improve the detection of melanoma and clinical outcomes for patients.

## Figures and Tables

**Table 1:** Heritable and nonmodifiable risk factors and their associated risk ratios for melanoma development.

Heritable Trait/Risk Factor	Risk Ratio	95% Confidence Interval
Common Nevi		
0–15	Reference	N/A
16–40	1.47	(1.36, 1.59)
41–60	2.24	(1.90, 2.64)
61–80	3.26	(2.55, 4.15)
81–100	4.74	(3.44, 6.53)
101–120	6.89	(4.63, 10.25)
Atypical Nevi		
0	Reference	N/A
1	1.60	(1.38, 1.85)
2	2.56	(1.91, 3.43)
3	4.10	(2.64, 6.35)
4	6.55	(3.65, 11.75)
5	10.49	(5.05, 21.76)
Phototype		
IV	Reference	N/A
III	1.77	(1.23, 2.56)
II	1.84	(1.43, 2.36)
I	2.09	(1.67, 2.58)
Eye Color		
Dark	Reference	N/A
Blue	1.47	(1.28, 1.69)
Green	1.61	(1.06, 2.45)
Hazel	1.52	(1.26, 1.83)
Hair Color		
Dark	Reference	N/A
Red	3.64	(2.56, 5.37)
Blonde	1.96	(1.41, 2.74)
Light Brown	1.62	(1.11, 2.34)
Family History		
No	Reference	N/A
Yes	1.74	(1.41, 2.14)

Data for Table 1 adapted from Gandini et al. 2005 a,b [19,21].

**Table 2:** A summary of the clinical evidence for chemopreventive agents.

Chemopreventive Agent	Proposed Mechanism of Action	Clinical Data	Evidence for Clinical Efficacy
Nicotinamide (oral)	Antioxidant, antiproliferative, reduces photoimmunosuppression	Chen et al., 2015—No change in incidence of melanomas. Decreased number of keratinocytic carcinomas (23% fewer) and actinic keratoses (13% fewer) than the placebo group.	Efficacy limited to keratinocyte carcinomas
Myristyl Nicotinate (topical)	Enhances skin cell turnover and epidermal differentiation	NCT00619060—Did not achieve safety endpoint in phase I clinical trial.	No demonstrable clinical efficacy
Celecoxib (oral)	Decrease potentially oncogenic prostaglandin synthesis and release	Elmets et al., 2010—43% lower risk of developing keratinocyte carcinomas in patients treated with celecoxib versus placebo. Tang et al., 2010—20% increase in annual basal cell carcinoma burden in treated patients versus 50% increase in placebo for patients with basal cell nevus syndrome.	Efficacy limited to keratinocyte carcinomas
Sulindac (oral)	Decrease potentially oncogenic prostaglandin synthesis and release	Lewandrowski et al., 2012—Sulindac metabolites were detected in atypical nevi; however, no significant changes in apoptosis or vascular endothelial growth factor A expression were noted.	No demonstrable clinical efficacy
Diclofenac (topical)	Decrease potentially oncogenic prostaglandin synthesis and release	Jeter et al., 2016—Diclofenac with or without DFMO increased markers of cutaneous inflammation (karyometric average nuclear abnormality (ANA)).	No demonstrable clinical efficacy
Aspirin (oral)	Decrease potentially oncogenic prostaglandin synthesis and release	Varedi et al., 2020—Dose reduction in prostaglandin E_2_ levels in melanocytic nevi after administration of aspirin for one week (50–70% decrease for 325mg cohort, 35–50% decrease for 81 mg cohorts).	Early clinical evidence of molecular effect
Isotretinoin (oral)	Anti-inflammatory and antiproliferative	Kraemer et al., 1988—Daily oral isotretinoin for two years reduced the incidence of new skin cancers in patients with xeroderma pigmentosum by 63%. Levine et al., 1997—Daily isotretinoin for three years resulted in no difference in keratinocyte carcinoma incidence compared to placebo in patients with a history of multiple keratinocyte carcinomas.	Efficacy limited to keratinocyte carcinomas
Acitretin (oral)	Anti-inflammatory and antiproliferative	Kadakia et al., 2012—Acitretin 25 mg orally 5 days per week for two years produced no significant difference in keratinocyte carcinoma incidence in the treatment arm versus placebo.	No demonstrable clinical efficacy
Tretinoin (topical)	Anti-inflammatory and antiproliferative	Weinstock et al., 2012—Tretinoin twice daily for 1.5-5.5 years produced no significant difference in keratinocyte incidence compared to placebo.	No demonstrable clinical efficacy
N-acetylcysteine (NAC) (oral)	Antioxidant	Cassidy et al., 2017—No significant difference in markers of UV-induced oxidative stress between NAC-treated and placebo groups.	No demonstrable clinical efficacy
Vitamin D (oral)	Antiapoptotic, photoprotective, reduces photoimmunosuppression	Scott et al., 2017—Patients treated with Vitamin D had decreased inflammatory changes following experimental sunburn.	Early clinical evidence of molecular effect
Statins (oral)	Antiproliferative, inhibition of the Ras signaling pathway	Linden et al., 2014—Lovastatin 40mg daily produced no significant differences in histopathologic markers of atypia, clinical atypia, nevus number, or molecular biomarkers of oncogenesis in atypical nevi between the treatment arm and placebo.	No demonstrable clinical efficacy
T4 endonucleases (topical)	Enables repair of dipyrimidine photo-mutations	Yarosh et al., 2001—T4N5 liposome lotion decreased the annual incidence rate of actinic keratoses and basal cell carcinomas in patients with xeroderma pigmentosa. Stoddard et al., 2017- Significant reduction in the number of new actinic keratoses observed in treated patients, persisting 12 weeks after treatment.	Efficacy limited to keratinocyte carcinomas
Difluoromethylornithine (DFMO) (oral)	Decreases epithelial polyamine levels (potentially tumorigenic)	Bailey et al., 2010—Orally dosed DFMO (500 mg/m^2^/day) produced no significant difference in total new keratinocyte carcinomas versus placebo over 4 to 5 years.	No demonstrable clinical efficacy
Grape Polyphenols (oral)	Photoprotective	Oak et al., 2021—California table grape powder (25 g, 3x per day) for two weeks significantly increased MED from baseline levels.	Early clinical efficacy in experimental sunburn models
Lycopene (oral)	Antioxidant	Rizwan et al., 2011—16-mg lycopene daily for 12 weeks versus control produced a significant increase in MED compared to placebo, as well as a reduction in matrix metalloprotease-1 expression and mtDNA 3895-bp deletions, as well as an increase in procollagen deposition.	Early clinical efficacy in experimental sunburn models
Selenium and Vitamin E (oral)	Antioxidant	Argos et al., 2017—Selenium and vitamin E supplementation (alpha-tocopherol (100 mg daily) and L-selenomethionine (200 μg daily) did not significantly reduce the incidence of keratinocyte carcinoma development over six years.	No demonstrable clinical efficacy
Vitamins E and C and Zinc (oral)	Antioxidant	Lloret et al., 2015—Supplementation with vitamin C 500 mg, vitamin E 400 IU, and zinc 50 mg per day did not significantly reduce levels of oxidative stress biomarkers between groups.	No demonstrable clinical efficacy
Omega-3 Polyunsaturated Fatty Acids (PUFA) (oral)	Reduces photoimmunosuppression	Pilkington et al., 2013—5-g omega-3 PUFA or a control lipid daily for 3 months did not produce a significant difference in measures of photoimmunosuppression in experimentally irradiated patients versus controls.	No demonstrable clinical efficacy
